# The chromosome-level genome of the submerged plant *Cryptocoryne crispatula* provides insights into the terrestrial–freshwater transition in Araceae

**DOI:** 10.1093/dnares/dsae003

**Published:** 2024-01-20

**Authors:** Zhi-Hao Qian, Wei Li, Qing-Feng Wang, Shi-Chu Liang, Shuang Wu, Zhi-Zhong Li, Jin-Ming Chen

**Affiliations:** Aquatic Plant Research Center, Wuhan Botanical Garden, Chinese Academy of Sciences, Wuhan 430074, China; University of Chinese Academy of Sciences, Beijing 100049, China; Aquatic Plant Research Center, Wuhan Botanical Garden, Chinese Academy of Sciences, Wuhan 430074, China; Plant Diversity Research Center, Wuhan Botanical Garden, Chinese Academy of Sciences, Wuhan 430074, China; Sino-Africa Joint Research Center, Chinese Academy of Sciences, Wuhan 430074, China; Key Laboratory of Ecology of Rare and Endangered Species and Environmental Protection (Guangxi Normal University), Ministry of Education, Guilin 541006, China; Guangxi Association for Science and Technology, Nanning 530023, China; Aquatic Plant Research Center, Wuhan Botanical Garden, Chinese Academy of Sciences, Wuhan 430074, China; Aquatic Plant Research Center, Wuhan Botanical Garden, Chinese Academy of Sciences, Wuhan 430074, China

**Keywords:** adaptation, *Cryptocoryne crispatula*, genomic synteny, mutation rates, terrestrial–freshwater transition

## Abstract

Plant terrestrialization (i.e. the transition to a terrestrial environment) is a significant evolutionary event that has been intensively studied. While certain plant lineages, particularly in angiosperms, have re-adapted to freshwater habitats after colonizing terrene, however, the molecular mechanism of the terrestrial–freshwater (T–F) transition remains limited. Here, the basal monocot Araceae was selected as the study object to explore the T–F transition adaptation mechanism by comparative genomic analysis. Our findings revealed that the substitution rates significantly increased in the lineage of freshwater Araceae, which may promote their adaptation to the freshwater habitat. Additionally, 20 gene sets across all four freshwater species displayed signs of positive selection contributing to tissue development and defense responses in freshwater plants. Comparative synteny analysis showed that genes specific to submerged plants were enriched in cellular respiration and photosynthesis. In contrast, floating plants were involved in regulating gene expression, suggesting that gene and genome duplications may provide the original material for plants to adapt to the freshwater environment. Our study provides valuable insights into the genomic aspects of the transition from terrestrial to aquatic environments in Araceae, laying the groundwork for future research in the angiosperm.

## 1. Introduction

About 500 million years ago, the common ancestor of land plants successfully overcame drought stresses and established diverse land plant communities that exist today.^1^ Despite the overall trend of plant life on land, certain plant groups have returned to aquatic habitats (freshwater and marine environments). Fossil and phylogenetic evidence suggests that angiosperms, dating back to the Cretaceous period, were already adapted to aquatic habitats.^2^ Aquatic angiosperms are present in approximately 17% of angiosperm families, yet they represent less than 2% of extant angiosperms, with at least 100 independent evolutionary origins.^3,4^ A recent study has revealed that the limited diversity of aquatic angiosperms today can be attributed to low diversification rates and infrequent transitions to water habitats.^5^ Adapting to aquatic habitats poses significant challenges for terrestrial angiosperms. In comparison to their terrestrial counterparts, aquatic angiosperms encounter unique environmental stresses, including reduced light, hypoxia, low carbon dioxide levels, and mechanical damage from waves.^6^ To survive in hypoxic conditions, aquatic plants have displayed several adaptive characteristics in their structure and metabolism, including the presence of well-developed aerenchyma in roots, stems, and leaves, as well as enhanced glycolytic fluxes and ethanol fermentation.^7,8^

Understanding the molecular mechanisms behind the evolution of angiosperms from terrestrial to aquatic habitats has been a topic of great interest among scientists. With the arrival of the genome era, researchers have started investigating the terrestrial-aquatic transition of angiosperms at the genomic level. Initially, the focus was on the transition from terrestrial to marine environments. For instance, the publication of five seagrass genomes provided valuable insights into the genomic adaptations associated with a marine lifestyle.^9–11^ Additionally, several studies have explored the transition from terrestrial to freshwater habitats by examining positive selection and identifying extended gene families from a genomic perspective. For example, Carretero-Paulet et al.^12,13^ sequenced the genome of the freshwater plant *Utricularia gibba* and identified key genes and gene families associated with its unique lifestyle and specialized body plan. These genes are involved in auxin metabolism, signal transduction, peptidases, plant morphogenetic/developmental pathways, and response to environmental stimuli. Fang et al.^14^ reported the genome of the floating plant *Landoltia punctata* and discovered the gradual reduction of stomatal development, root development, lignin content, phytohormone pathways, and their corresponding genes in duckweed. However, due to the morphological reduction and simplified genomes of *U*. *gibba* and duckweed, these studies may not provide a comprehensive understanding of plant adaptation to freshwater habitats, which hinders a deeper exploration of the molecular mechanisms underlying the terrestrial–freshwater (T–F) transition. Additionally, it has been demonstrated that molecular evolutionary rates are associated with species’ morphological evolution and adaptation to specialized habitats.^15,16^ For instance, increased rates of molecular evolution are a common pattern of adaptation for parasitic plants and those living in rapid streams.^17,18^ Moreover, Nauheimer et al.^19^ found high mutation rates in aquatic plants within the family Araceae. Therefore, it is worth exploring whether changes in mutation rates are related to the adaptation of aquatic plants to their aquatic habitats.

In plants, extensive genome replication events have been found to be associated with adaptation to extreme environments and successful colonization of new areas.^20,21^ For instance, polyploidy events have been found to enhance adaptation to high salinity and drought stress in limestone karst for *Primulina huaijiensis*.^22^*Arabidopsis thaliana* polyploids have also shown increased potassium uptake and salinity tolerance.^23^ However, exploring the mechanisms by which gene and genome duplications contribute to evolutionary innovation remains a challenging task, and it is still unclear whether gene and genome duplications play a role in adaptation to aquatic environments.

Araceae is an early diverging monocot clade that comprises approximately 3,600 species in 144 genera worldwide.^24,25^ These plants occupy diverse habitats and exhibit a remarkable diversity of life forms, including geophytes, climbers, epiphytes, submerged plants, and free-floating aquatics.^24,26^ Fossils from the Upper Cretaceous and Paleocene periods provide evidence of floating Araceae, suggesting that the transition from terrestrial to freshwater habitats occurred early in the family evolution.^27,28^ Furthermore, multiple independent T–F transitions have occurred within Araceae. For example, a free-floating habit evolved for a third time in the ancestor of the monotypic genus *Pistia* within Araceae.^28^ Therefore, the Araceae family is an ideal group for studying the evolution of angiosperms from terrestrial to freshwater habitats and the differentiation of life forms. Although some genomes of Araceae have been published, such as those of floating plants *P. stratiotes*,^29^*Spirodela polyrhiza*,^30^ and terrestrial plants *Colocasia esculenta*,^31^*Pinellia pedatisecta*,^32^ there is a lack of genomic resources for submerged plants, which hinders further exploration of T–F transitions in Araceae.

In this study, we present a high-quality genome assembly of *Cryptocoryne crispatula*, a submerged plant belonging to the family Araceae. Furthermore, we conducted a comparative analysis of freshwater and terrestrial plant genomes within Araceae. We examined T–F transitions from multiple perspectives, such as gene family expansion and contraction, evolutionary rates, positive selection, and gene and genome duplications. Our results provide valuable insights into the genetic mechanisms of transitioning from terrestrial to freshwater environments.

## 2. Materials and methods

### 2.1. Sampling and genome size estimation

The sequenced sample of *Cryptocoryne crispatula* (2*n* = 32) was collected at the Wuhan Botanical Garden, Chinese Academy of Sciences in Hubei Province, China. High-quality DNA was extracted from fresh young leaves of the plant using the MagicMag Plant Genomic DNA Micro Kit (Sangon Biotech Co., Shanghai, China).

To estimate the genome size of *C*. *crispatula*, flow cytometry was applied with the *Nelumbo nucifera* (genome size = 808 Mb) as a reference.^33^ Also, *k*-*mer* analysis was performed on Illumina paired-end short reads using Jellyfish v2.1.3.^34^ The heterozygosity and repeat content were estimated using GCE v1.0.0 (https://github.com/fanagislab/GCE) based on *k-mer* = 17.

### 2.2. Genome sequencing and *de novo* genome assembly

Three sequencing strategies (Illumina, Nanopore, and Hi-C sequencing) were utilized in this study to sequence the genome. The paired-end 150 bp (PE150) read sequencing libraries were established on an Illumina NovaSeq 6000 platform with an insert size of 350 bp. Nanopore libraries were constructed using the Oxford Nanopore LSK-109 kit, following the standard protocol. These libraries were then sequenced on the PromethION platform, using R9.4.1 Spot-On Flow Cells chemistry and guppy software. The Hi-C libraries were constructed and sequenced on the Illumina HiSeq 2500 platform (Illumina, San Diego, CA, USA), using paired-end 125 bp mode, following a standard procedure described previously.^35^

The *C*. *crispatula* genome was *de novo* assembled based on Nanopore long reads, using NextDenovo v2.5.0 (https://github.com/Nextomics/NextDenovo), with default settings, except for the read_cutoff set to 28,666. The contigs were polished in three rounds, using Illumina short reads and Nanopore long reads, using NextPolish v1.4.0^36^ with default parameters. Subsequently, the software Purge_dups v1.2.5^37^ was employed to remove haploids and contig overlaps from the initial genome. Based on Hi-C data, ALLHiC v0.8.12^38^ was used to improve the draft genome to chromosome-level assembly based on chromosomal Hi–C interactions. Finally, JuiceBox v1.8.8^39^ was applied to visualize the Hi–C map and manually correct any assembly errors. In addition, the integrity of the genome assembly was assessed using BUSCO v5.4.7^40^ with the embryophyta_odb10 database. Merqury v1.3^41^ was utilized to estimate the consensus quality values (QV) of genome assembly based on Illumina short-reads.

### 2.3. RNA extraction and transcriptome sequencing

Six tissues from *C*. *crispatula*, including leaf, bract, pistil, stamen, fruit, and root, were collected for transcriptome sequencing. The total RNA of each tissue was isolated using the plant RNA isolation kit (DP432; Tiangen Technologies, Beijing, China). The RNA-seq libraries were constructed using the Truseq Stranded RNA Library Prep Kit (Illumina) and sequenced on the NovaSeq 6000 platform with PE150 mode.

### 2.4. Repeat element identification and genome annotation

The *de novo* repeat library of the *C*. *crispatula* assembly was first constructed using RepeatModeler v2.0.1^42^ with default parameters. Then, RepeatMasker v4.0.7 (http://www.repeatmasker.org) was used to search repeat elements based on the *de novo* repeat library and the RepeatMasker database (http://www.repeatmasker.org).

For protein-coding gene prediction analysis in the *C*. *crispatula* genome, a combination of *ab initio* and homology- and transcriptome-based pipelines was employed. The *ab initio* annotation was performed using Augustus v3.3.3^43^ with default parameters. The homology-based annotation utilized GeMoMa v1.7.1^44^ with protein sets from five closely related species: *Zostera marina*, *P*. *stratiotes*, *S*. *polyrhiza*, *C*. *esculenta*, and *Oryza sativa*. The transcriptome-based annotation involved two strategies: (i) mapping the RNA-seq data to the *C*. *crispatula* genome using HISAT2 v2.2.1,^45^ followed by transcripts assembled using StringTie v1.3.3^46^ and predicting open reading frame within transcripts using TransDecoder v5.5.0 (https://github.com/TransDecoder/TransDecoder); (ii) assembling the RNA-seq data using Trinity v2.8.5,^47^ and predicting gene structures using PASA v2.4.1.^48^ The results of all these predictions were integrated using EVM v1.1.1,^49^ with PASA used to update the EVM results.

Functional annotation of the protein-coding genes was performed using BLASTP v2.2.31 (*E*-value = 1E−05) against six protein databases: GO,^50^ KEGG,^51^ KOG (https://ftp.ncbi.nih.gov/pub/COG/KOG/), NR (https://ftp.ncbi.nlm.nih.gov/), InterPro,^52^ and Swiss-Prot (http://www.expasy.ch/sprot) databases.

### 2.5. Phylogenetic and gene family analyses

Seven Araceae plants, including three terrestrial plants (*A. konjac, C. esculenta,* and *P. pedatisecta*) and four freshwater plants (*C. crispatula, P. stratiotes*, *L. minuta*, and *S. polyrhiza*), and *Acorus tatarinowii* was selected as outgroup for gene family clustering using Orthofinder v2.5.2^53^ (Supplementary Table S1). MUSCLE v3.8^54^ was utilized to align the protein sequences of single-copy orthologous genes and convert the alignments into codon sequences by PAL2NAL v14.^55^ Then, Gblocks v0.91b^56^ was used to filter out non-conservative regions. The filtered sequences were then concatenated, and a phylogenetic tree was constructed using IQ-TREE v2.1.2 under the GTR+I+G4+F substitution model with 1000 bootstrap replicates.^57^ The divergence time was estimated using the MCMCtree program in PAML v4.9.^58^ The crown times of Araceae (107–114 Mya),^59^ the divergence times of *C*. *esculenta*–*P*. *pedatisecta* (17–41 Mya), and *Lemna minuta*–*S*. *polyrhiza* (44–73 Mya) from the TimeTree database (http://www.timetree.org/) was set for calibration. CAFE v5.1^60^ was employed to detect gene family size changes in each species, and the expansion and contraction of gene families at each branch were determined with a *P*-value < 0.05. GO and KEGG pathway enrichment analysis was performed using OmicShare tools (https://www.omicshare.com/tools).

### 2.6. Analysis of codon substitution rate associated with freshwater adaptation

For the convenience of function annotation, we generated additional datasets, including seven Araceae species mentioned in Section 2.5 and A. *thaliana* using Orthofinder. A total of 6,464 orthologous groups (Ogs) were identified. Then, the codon alignments of the 6,464 OGs were obtained using MUSCLE, PAL2NAL, and Gblocks. To investigate the association between the rate of plant molecular evolution and freshwater adaptation, we applied the free-ratio model to calculate the non-synonymous substitution rate (*dN*) and synonymous substitution rate (*dS*) separately for each species using the model of codeml in PAML v4.9 with default parameters.^58^ We excluded data with *dS* <0.0005 or *dS* >2 due to the possibility of errors or pseudogenes in multiple sequence comparisons.^61^ In addition, the significant association of substitution rates between freshwater and terrestrial plants was examined by Wilcoxon signed ranks test in SPSS v21.0.

### 2.7. Identification of positively selected genes

To test for the presence of positive selection in freshwater species, we employed the branch-site model implemented in the codeml module of PAML. Each freshwater species was designated as a foreground branch in this analysis based on the 6464 OGs. The null model assumed that foreground sites were either neutral or under purifying selection, while the alternative model allowed for positive selection at these sites. Likelihood ratio tests (LRT) were conducted to compare the alternative and null models. Differences in LRT results were evaluated using Chi-square tests with 2 degrees of freedom, and a significance level of *P*-value < 0.05 was considered.

### 2.8. Collinearity analysis

The R package *syntenet*^62^ can be used to infer and analyze synteny networks based on whole-genome protein sequence data. Synteny networks inferred using *syntenet* can identify taxon-specific gene clusters that likely played a role in the evolution of important traits.^63^ In this study, we utilized the ‘find_GS_clusters()’ function in the R package *syntenet* to identify specific synteny gene clusters in freshwater and terrestrial plants, respectively. Also, submerged plant-specific synteny clusters and floating plant-specific synteny clusters were identified based on terrestrial, floating, and submerged plants as the basis for grouping, respectively. GO and KEGG pathways of freshwater plant-specific genes and floating plant-specific genes were assigned according to the orthologous genes of the *C*. *crispatula* genome and *S*. *polyrhiza* genome, respectively.

## 3. Results and discussion

### 3.1. Chromosome-level genome assembly of *C. crispatula*

The size of the *C*. *crispatula* genome was estimated to be approximately 886 Mb using flow cytometry (Supplementary Fig. S1). Further *k-mer* analysis revealed a genome size estimate of 836 Mb with high heterozygosity (1.03%; Supplementary Table S2; Supplementary Fig. S2). Additionally, we obtained approximately 253 Gb (approximately 316× sequence depth) of Illumina paired-end reads and approximately 123 Gb (approximately 155× sequence depth) of Nanopore long reads (Supplementary Table S3). These reads were then assembled into a draft genome of approximately 801.62 Mb, consisting of 470 contigs with an N50 value of 3.85 Mb and the longest contig measuring 14.94 Mb (Table 1). Furthermore, approximately 98.80% of the contigs (~792.08 Mb) were anchored to 18 pseudochromosomes using approximately 102 Gb of Hi-C clean reads (Supplementary Table S4; Supplementary Fig. S3). The final chromosome-level genome assembly of *C*. *crispatula* was 801.66 Mb with a scaffold N50 length of 43.92 Mb (Table 1). The BUSCO assessment revealed that *C*. *crispatula* genome had the highest completeness score (97.0%) of BUSCO gene models in freshwater Araceae, compared with 86.3% of *L. minor*, 90.0% of *P. stratiotes* and 94.0% of *S. polyrhiza* (Supplementary Fig. S4). Moreover, the QV score (30.42) of the *C*. *crispatula* genome was >30 (Table 1). Thus, these results supported that the *C*. *crispatula* genome was nearly complete and highly accurate.

**Table 1.** Statistics of *C. crispatula* genome assembly

Assembly	
Nanopare sequencing assembly	
Number of sequences	470
Total length (bp)	801,624,686
N50 length (bp)	3,851,604
Longest contig	14,940,495
Hi-C assembly	
Number of sequences	79
Total length (bp)	801,664,786
N50 length (bp)	43,917,102
Longest scaffold	58,176,801
Number of contigs not in scaffolds	61
Total_N_counts	41,319
GC percentage	40.70%
Percentage of repeats	68.43%
QV	30.42

### 3.2. Genome annotation

**Figure 1.**
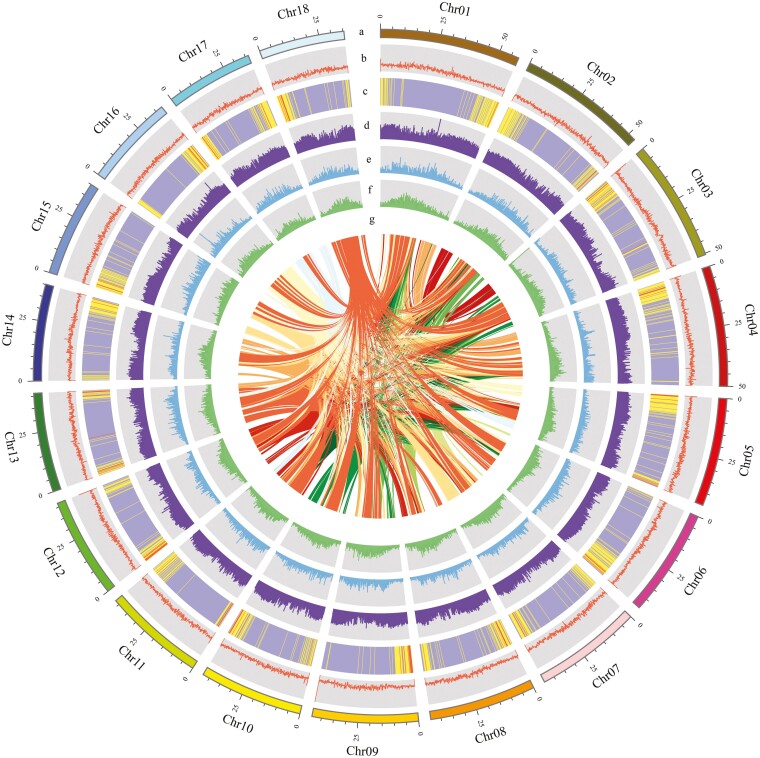
An overview of genomic features *Cryptocoryne crispatula* genome. (a) Chromosome length; (b) GC content; (c) gene density; (d) repeat coverage; (e) LTR-*Gypsy* density; (g) LTR-*Copia* density; (g) syntenic blocks. The window size used in the circles was 200 kb.

Approximately 68.43% of repetitive contents were identified in the genome of *C*. *crispatula*. Among these repetitive elements, approximately 29.74% were LTR retrotransposons, including LTR-*Gypsy* (20.88%) and LTR-*Copia* (8.26%; Supplementary Table S5). Also, a total of 30,010 protein-coding genes were predicted, with an average coding sequence length of 280.1 bp and an average of 5.1 exons per gene (Supplementary Table S6). We observed that the distribution of genes was uneven, with a higher concentration at the chromosomal ends (Fig. 1), similar to the close relative species *P. stratiotes*.^32^ Among the protein-coding genes, 28,694 (95.61%) were functionally annotated using six public databases. Notably, 92.62% of the genes had a match in the NR database (Supplementary Table S7). To validate our gene annotation results, we assessed the completeness of the gene sets using BUSCO analysis. The analysis showed that 95.2% of the complete BUSCOs were present, indicating the reliability of our annotation results (Supplementary Table S8). Additionally, 652 tRNAs, 148 rRNAs, 90 snRNAs, and 14 miRNAs were predicted in the *C*. *crispatula* genome (Supplementary Table S9).

### 3.3. Expanded and contracted gene families

**Figure 2.**
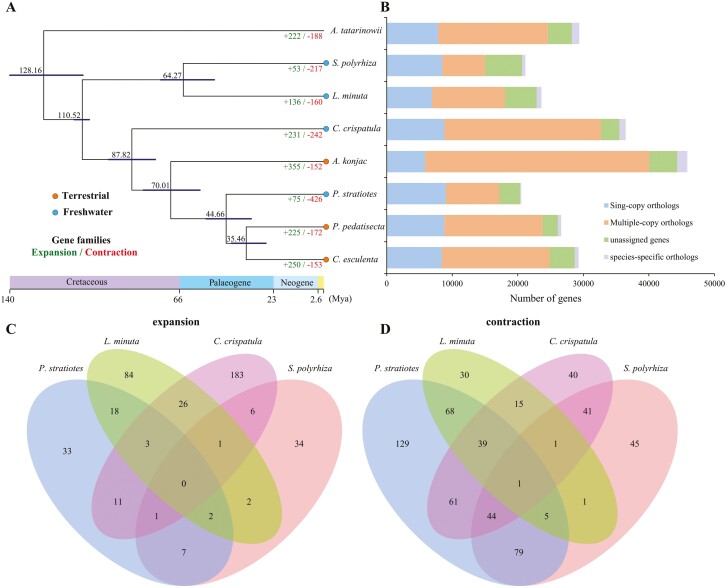
Gene family analysis. (A) Phylogenetic tree showing divergence times and the evolution of gene families in freshwater Araceae. The estimated divergence times (million years ago, Mya) are shown at each node. Expansion and contraction of gene families are denoted as numbers with green and red, respectively. (B) Bar plot showing gene number identified by OrthorFinder. Unassigned genes: a gene that has not been assigned any orthogroup, i.e. singleton genes that have no orthologs in other species and no copy genes within a species. (C) Venn diagram showing the expanded gene families among four freshwater plants. (D) Venn diagram showing the contracted gene families among four freshwater plants.

This study used 756 common single-copy orthologous genes among eight species (*A*. *tatarinowii* as the outgroup) to infer the phylogeny and estimate divergence time. The phylogenetic tree revealed at least three independent origins of freshwater species within Araceae (Fig. 2A), consistent with previous findings.^19^ Through gene family clustering analysis, we found that 196,172 genes (86.5%) were clustered into 22,093 OGs (Fig. 2B). Among these four freshwater species, the number of significantly contracted gene families was much larger than the number of significantly expanded gene families. Here, we considered gene families with significant expansion or contraction in over three freshwater species as gene families associated with expansion or contraction in freshwater species. In total, seven significantly expanded gene families and 90 significantly contracted gene families were identified in freshwater species (Fig. 2C and D). These genes were subjected to GO and KEGG enrichment analysis and were found to be overrepresented in several major molecular functions. The enriched pathways of the expanded gene families were primarily related to basal metabolism and biosynthesis pathways, such as the phosphorus metabolic process, pentose and glucuronate interconversions, zeatin biosynthesis, and carbohydrate metabolic process (Supplementary Table S10). The latter category included many enzymes involved in pectin biosynthesis, commonly associated with different carbohydrate metabolic pathways in plant-type cell walls. Examples of such enzymes include polygalacturonases of OG0010560 (Supplementary Table S11), which have been shown to play roles in various plant developmental programs, including fruit and anther dehiscence, seed germination, pollen tube growth, and organ abscission.^64^ In addition, the expansion of multicopper oxidases of OG0000153 occurred (Supplementary Table S11, Supplementary Fig. S5), including *LOW PHOSPHATE ROOT1* (*LPR1*) and *LOW PHOSPHATE ROOT2* (*LPR2*) that together with a P5-type ATPase in a common pathway that adjusts root meristem activity to inorganic phosphate (Pi) availability.^65^ Previous studies have also found that aquatic- or wetland-related plants had higher copy numbers of this gene family than terrestrial plants,^66^ indicating that the expansion of *LPR1/LPR2* may have played a key role in the adaptation of freshwater habitats with low Pi availability. So, it is tempting to speculate that the expansion of gene families associated with basal metabolism may serve as the genetic basis for T–F transition. On the other hand, the enriched pathways of the contracted gene families were primarily involved in signal transduction and defense against pathogens, including GO terms such as enzyme-linked receptor protein signaling pathway, defense response to oomycetes, and defense response to bacteria (Supplementary Table S12). Related gene families include serine threonine-protein kinase (Supplementary Table S13), which are implicated in pathogen tolerance and sensing extracellular ATP,^67^ plant mitogen-activated protein kinases that play vital roles in plant defense responses and signal transduction,^68^ and leucine-rich repeat receptor kinases (LRR-RKs), which are involved in cell death control and pathogenesis.^69^ Additionally, previous research has shown that gene families associated with disease resistance undergo convergent reduction in aquatic plants.^70^ The microbial composition of the rhizosphere may vary between aquatic and terrestrial plants due to the anaerobic conditions present around the roots of aquatic plants. Therefore, the altered or reduced interactions between microbes and the roots of aquatic plants may imply the absence of certain immune responses in aquatic plants.

In general, freshwater plants-specific genes are likely to be vital for T–F transition. Here, we identified freshwater plants-specific OGs, and found that the 3 OGs were shared only among freshwater species (Supplementary Table S14). Intriguingly, only OG0015158 could annotate to the function that belongs to the L-Lactate/malate dehydrogenases (LDH/MDH) superfamily. The LDH/MDH gene families are critical in the adaptation of animals to aquatic environments with pressure and hypoxic.^71^ In plants, some members of this gene family could increase root hypoxia and low Pi stress tolerance,^72,73^ suggesting that the LDH/MDH gene families may play vital roles in the adaptation of freshwater habitats with low Pi and hypoxia.

### 3.4. High substitution rates in freshwater Araceae

**Figure 3.**
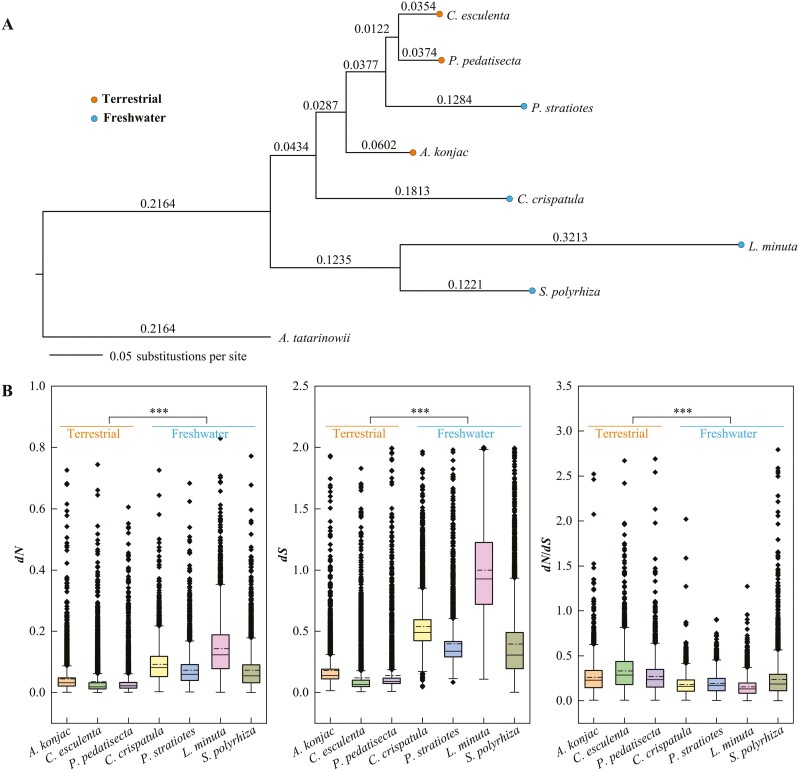
Comparative analysis of evolutionary rates between freshwater and terrestrial plants. (A) A maximum-likelihood tree was inferred using IQ-TREE, with *A. tatarinowii* as an outgroup. The number on the branch represents the branch length information. (B) We applied the free-ratio model to calculate *dN*, *dS,* and *dN/dS* separately for each species. The dotted line of the box plot means the average value. *P*-value is from Wilcoxon signed ranks test (***P < 0.001).

In the phylogenetic analysis, we discovered that the branch lengths of four freshwater plants were longer than those of terrestrial plants, indicating higher DNA substitution rates in the freshwater species (Fig. 3A), consistent with previous findings.^19^ Since synonymous mutations are considered nearly neutral, mutation rates are often assessed by examining *dS*.^74^ Therefore, the free-ratio model was used to calculate *dN* and *dS* separately for each species using 6,464 OGs to determine if the mutation rates were elevated in the four freshwater species. After excluding data with *dS* < 0.0005 and *dS* > 2, we were left with 3,966 OGs. We observed that both *dN* and *dS* values for freshwater plants were significantly higher than those for terrestrial plants, indicating increased mutation rates in freshwater plants (Wilcoxon signed ranks test, *P*-value < 0.001, Fig. 3B and C). Interestingly, the ratio of *dN*/*dS* was significantly lower for freshwater plants compared with terrestrial plants, which may be attributed to the greater increase in *dS* for terrestrial plants (*P*-value < 0.001, Fig. 3D). Additionally, our analysis revealed that 98.5% of genes on the freshwater lineage had a *dN*/*dS* ratio smaller than 1, suggesting that purifying selection has been active at the molecular level throughout the evolutionary history of freshwater plants. To investigate the factors causing high mutation rates in freshwater plants, according to the method of Schumacher and Herlyn,^75^ we divided the 3,966 OGs into three equal parts (‘low *dS*’, ‘medium *dS*’, and ‘high *dS*’) based on *dS* estimates (Supplementary Table S15). We only focus on genes with high mutation rates, referred to as ‘high *dS*’, and found the 271 OGs with ‘high *dS*’ were shared in four freshwater plants (Supplementary Fig. S6). Further, we conducted GO and KEGG enrichment analysis for the 271 OGs with ‘high *dS*’. These genes were primarily involved in transcriptional regulation and signal transduction at a functional level (Supplementary Fig. S7).

DNA substitutions are commonly regarded as observable mutations when analyzing DNA sequence data.^76^ The causes of rapid protein evolution in freshwater Araceae are still unclear. Previous studies have suggested that variations in mutation rates in both animals and plants can be attributed to various factors, including differences in body size, metabolic rate, life-history traits, environmental energy, DNA repair, generation times, and lifestyle.^75,77,78^ In our study, we found that many genes with a high mutation rate were functionally related to transcriptional regulation, such as regulation of transcription, DNA-templated, regulation of nucleic acid-templated transcription, and regulation of RNA biosynthetic process, among others (Supplementary Fig. S7). These pathways included transcription factors like *ARABIDOPSIS NAC DOMAIN CONTAINING PROTEIN 2* (*ANAC2*) and *EIN2 NUCLEAR ASSOCIATED PROTEIN 1* (*ENAP1*) (Supplementary Table S16), both of which have been shown to regulate drought response in *A*. *thaliana*.^79,80^ Additionally, it has been reported that the response of plant cells to a low-oxygen environment under waterlogging stress involves adjusting energy expenditure and complex regulation of gene expression at the transcriptional synthesis level.^81–83^ For example, genes associated with transcriptional regulatory pathways were up-regulated under flooding stress in *Brassica napus*.^83^ Furthermore, genes with a high mutation rate were found to be significantly enriched in signal transduction pathways, such as MAPK signaling pathway – plant and plant hormone signal transduction, among others (Supplementary Fig. S7). Examples include the negative regulator *PROTEIN PHOSPHATASE 2CA* (*PP2CA*) of abscisic acid (ABA) signaling, which activates the transcription factor *WRKY33*, and the auxin-responsive *GH3* gene family (Supplementary Table S16, Supplementary Fig. S8). Consistently, phytohormones mediate abiotic stress responses.^84^ The ubiquitin E3 ligase *SR1* regulates submergence response by degrading the transcription factor *WRKY33* in *A*. *thaliana*.^85^ In some aquatic plants, the rate of ethylene synthesis leads to a decrease in ABA levels, which increases sensitivity to gibberellin (GA) and ultimately stimulates internode growth under waterlogging conditions.^86^ In addition, OG0001726, OG0002393, and OG0006038 belong to LRR-RKs containing bacterium defense-related that are involved in the immune response of freshwater plants through elevated mutation rates (Supplementary Table S16). These results suggest that transcriptional regulation and signal transduction play a crucial role in adaptation to water. Therefore, the high mutation rate in freshwater Araceae may be related to their lifestyle, specifically their adaptation to freshwater habitats.

### 3.5. Detection of positive selection

In this study, we analyzed positive selection using the branch-site model on 6464 OGs. Among them, the 20 OGs were identified as positively selected genes (PSGs) and were found to be shared among all freshwater species (Supplementary Table S17, Supplementary Fig. S9). These PSGs are likely associated with adaptation to freshwater conditions. Functional enrichment analysis revealed that these PSGs were enriched for 101 GO terms and four KEGG pathways. Interestingly, several previously unreported terms related to adaptation to freshwater environments were identified (Supplementary Table S18). For instance, terms related to DNA repair, such as base-excision repair, DNA repair, and cellular response to DNA damage stimulus, were found to be enriched. Examples of genes associated with these terms include *REPRESSOR OF SILENCING 1* (*ROS1*), which is involved in active DNA demethylation,^87^ and *BREAST CANCER 2 LIKE 2A* (*BRCA2A*), which is required for somatic and meiotic homologous recombination.^88^ Furthermore, some PSGs were significantly enriched in pathways related to tissue development, including epidermis development, single-multicellular organism process, and epithelial cell differentiation. Specifically, *AUXIN-INDUCED IN ROOT CULTURES 3* (*AIR3*) has been shown to be up-regulated to facilitate lateral root emergence.^89^*EXOCYST COMPLEX COMPONENT SEC5* (*SEC5A*), a member of the exocyst complex gene family, is involved in primary root growth, pollen development, and polar growth of the pollen tube.^90,91^*TRANSPARENT TESTA GLABRA 2* (*TTG2*) is a key regulator of the differentiation of seed coat cells and is involved in the biosynthesis of seed mucilage and cuticle, including wax ester, in developing seeds.^92^*HOMEODOMAIN GLABROUS2* (*HDG2*) is a key epidermal component that promotes stomatal differentiation.^93^ The rapid evolution of these genes may contribute to the adaptation of freshwater plants to aquatic habitats, particularly in terms of root, pollen, and cell wall development. Moreover, several genes involved in the regulation of defense response (Supplementary Table S17), such as *ESSENTIAL FOR POTEXVIRUS ACCUMULATION 1* (*EXA1*),^94^*SECRETED ASPARTIC PROTEASE 1*,^95^*HDS-ASSOCIATED RLK1* (*HAK1*),^96^ and *BRCA2A*^88^ were found to have undergone positive selection. This suggests that immune regulation may be an important strategy for freshwater plants adaptation to freshwater habitats.

### 3.6. Syntenic genes specific to freshwater plants

**Figure 4.**
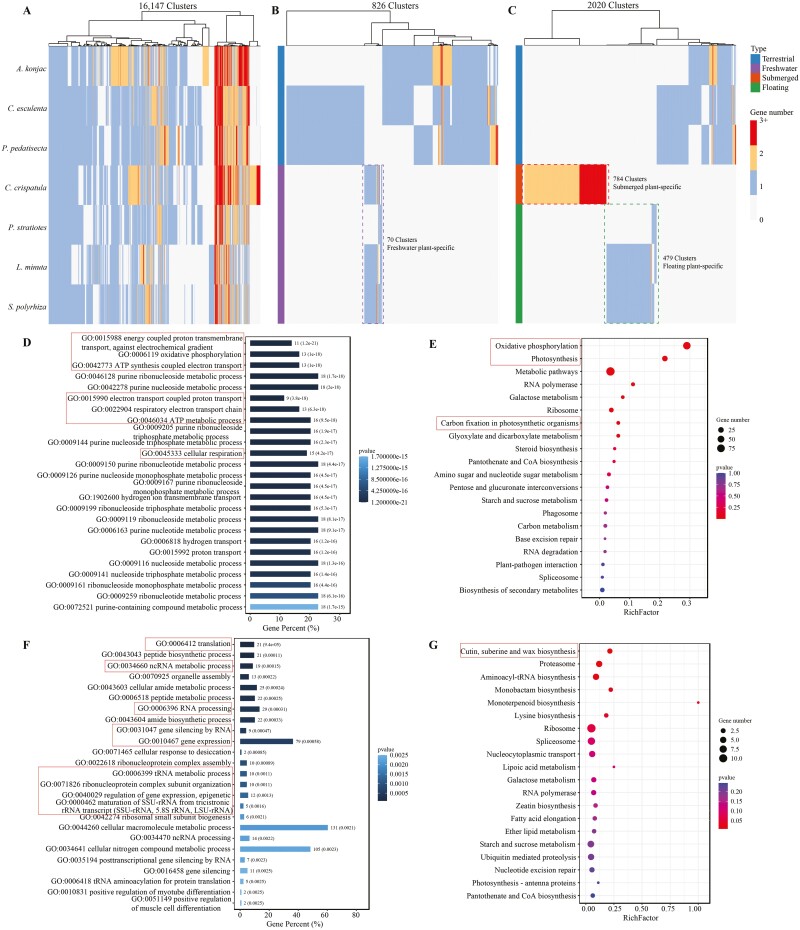
Hierarchical clustering of synteny genes in Araceae. (A) 16,147 synteny network clusters were identified in seven Araceae plants. (B) 70 freshwater plant-specific synteny clusters were identified in seven species. (C) 784 submerged plant-specific synteny clusters and 479 floating plant-specific synteny were identified in seven species, respectively. (D) Significantly enriched biological process top 25 GO terms of specific synteny clusters in submerged plant genomes. (E) Top 20 of KEGG enrichment of specific synteny clusters in submerged plant genomes. (F) Significantly enriched biological process top 25 GO terms of specific synteny clusters in floating plant genomes. (G) Top 20 of KEGG enrichment of specific synteny clusters in floating plant genomes.

Next, we investigated whether gene duplication played a role in the transition from terrestrial to freshwater environments. To do this, we utilized the R package syntenet to analyze synteny clusters specific to freshwater plants. We identified 16,147 synteny network clusters in seven Araceae species (Fig. 4A). Among these clusters, we found 70 freshwater plant-specific synteny clusters (Fig. 4B). We then conducted GO and KEGG enrichment analysis on these genes and discovered that they are primarily involved in the biosynthesis of various plant secondary metabolites, such as cyanoamino acid metabolism, pentose and glucuronate interconversions, and folate biosynthesis (Supplementary Table S19). Additionally, we extracted 784 submerged plant-specific synteny clusters (SPSCs) and 479 floating plant-specific synteny clusters (FPSCs) (Fig. 4C). The genes within the SPSCs were mainly enriched in cellular respiration (e.g. ATP synthesis coupled electron transport, oxidative phosphorylation, ATP metabolic process, respiratory electron transport chain), purine metabolism (e.g. purine ribonucleoside metabolic process, purine ribonucleoside triphosphate metabolic process), and photosynthesis (e.g. photosynthesis, carbon fixation in photosynthetic organisms) (Fig. 4D and E). On the other hand, the genes within the FPSCs were primarily enriched in the regulation of gene expression (e.g. RNA processing, gene silencing by RNA, gene expression) and biosynthesis processes (e.g. peptide biosynthetic process, cutin, suberine and wax biosynthesis, aminoacyl-tRNA biosynthesis, and monobactam biosynthesis) (Fig. 4F and G).

The extensive genome duplication events during evolution have been crucial in the adapting of species to new habitats.^21^ For instance, after the genome duplication event during the Cretaceous–Paleogene extinction period, duplicated genes contained key genes for adapting to extreme environmental changes such as cold and darkness. This suggests that genome duplications have enhanced the ability of plants to adapt to environmental stress.^97^ Most aquatic environments are shaded due to reflection loss at the air–water interface and light absorption by water and suspended or dissolved materials.^98,99^ The availability of CO_2_ in water is also reduced, which can limit photosynthesis.^100^ Therefore, submerged plants face unique stresses, including low light, reduced carbon availability, and sediment anoxia, compared with terrestrial, emergent, and floating plants.^101^ In response to these conditions, enhanced glycolytic flux and ethanol fermentation are often observed in aquatic plant metabolism.^7^ Our study showed that submerged plant-specific synteny genes associated with cellular respiration and photosynthesis underwent expansion. Notable examples include important subunits (*psaA* and *psaB*) of the photosystem photosynthetic system I, NADH dehydrogenase (e.g. *ND1*, *ND4*), and cytochrome c oxidase (*COX1* and *COX2*) (Supplementary Table S20, Supplementary Figs. S10 and S11). The increase in the number of copies of these genes may have enhanced the adaptation of submerged plants to the submerged environment.

In addition, we found that floating plant-specific synteny genes were enriched in gene regulatory pathways. For example, pentatricopeptide repeat (PPR) proteins (Supplementary Table S21), which bind to organelle transcripts, were identified as influencing gene expression by altering RNA sequence, turnover, processing, or translation.^102^ The expansion of related gene families suggests that floating plants can regulate transcription, allowing them to adapt flexibly to freshwater habitats. This finding is consistent with recent research on duckweed.^14^ Additionally, the primary role of the cuticle, composed of cutin and cuticular waxes, is to act as a physical barrier against UV radiation, pathogen attack, and mechanical damage.^103,104^ Since free-floating plants are exposed to direct sunlight, the expansion of genes associated with cutin, suberine, and wax biosynthesis may enhance their antibacterial ability and resistance to UV radiation. This may explain why duckweed and *P*. *stratiotes* can thrive in eutrophic wastewater. In summary, gene and genome duplications contribute to the adaptation of submerged and floating plants to different life forms.

## 4. Conclusions

In this study, we comprehensively analyzed the T–F transition in Araceae in terms of gene families, evolutionary rates, positive selection, and gene and genome duplication. We found that *LPR1/LPR2* genes and LDH/MDH gene families may contribute to the adaptation of freshwater plants to freshwater habitats with low Pi availability and hypoxia. In addition, our findings suggest that genes with high mutation rates in freshwater species of Araceae may be related to the environmental stresses from freshwater lifestyles, and these genes are adapted to freshwater habitats mainly through their involvement in transcriptional regulation and signal transduction pathways. Furthermore, 20 gene sets among all four freshwater species showed signs of positive selection, contributing to tissue development and defense responses in freshwater plants. Our synteny analyses demonstrate that gene and genome duplications enhance the adaptation of submerged and floating plants to different life forms. Overall, T–F transition for plants may result from the integrative influence of different processes. Also, our study provides new insights into the molecular mechanisms underlying plant transition from terrestrial to freshwater environments. However, it is essential to note that our study has only studied a few freshwater plant groups. In the future, it would be beneficial to integrate more genomic data from aquatic plant lineages and their terrestrial relatives to elucidate the molecular mechanisms of terrestrial-aquatic evolution further.

## Supplementary Material

dsae003_suppl_Supplementary_Tables

dsae003_suppl_Supplementary_Figures

## Data Availability

All the data sets (Illumina, Nanopare, Hi-C, RNA-seq and the genome assembly and annotation files) has been deposited at the China National GeneBank DataBase (CNGBdb, https://db.cngb.org/) website under the accessions CNS0855523- CNS0855527 with CNGB-Project ID CNP0004340. The assembled genome sequences were also deposited into NCBI GenBank under accession number JAYDZW000000000. The supplementary data sets of expanded gene families, contracted gene families, positively selected genes, genes with high mutation rates, freshwater plant-specific synteny genes floating plant-specific synteny genes, and submerged plant-specific synteny genes have been deposited into the Figshare database (https://doi.org/10.6084/m9.figshare.24782616.v1).
