# Supplementary material for: The chromosome-level genome of the submerged plant Cryptocoryne crispatula provides insights into the terrestrial–freshwater transition in Araceae
Source: DNA Res. 2024 Jan 20;31(1):dsae003. doi: 10.1093/dnares/dsae003 (PMC10873505; doi:10.1093/dnares/dsae003)
Supplement: dsae003_suppl_Supplementary_Figures [file dsae003_suppl_supplementary_figures.docx]

**Supplementary Figures for:**

**The chromosome-level genome of the submerged plant *Cryptocoryne crispatula* provides insights into the terrestrial-freshwater transition in Araceae**

Zhihao Qian^1,2^, Wei Li^1^, Qingfeng Wang^3,4^, Shichu Liang^5^, Shuang Wu^6^, Zhizhong Li^1^*, Jinming Chen^1^*

***^1^*** *Aquatic Plant Research Center, Wuhan Botanical Garden, Chinese Academy of Sciences, Wuhan 430074, China*

***^2^*** *University of Chinese Academy of Sciences, Beijing 100049, China*

***^3^*** *Plant Diversity Research Center, Wuhan Botanical Garden, Chinese Academy of Sciences, Wuhan 430074, China*

***^4^*** *Sino-Africa Joint Research Center, Chinese Academy of Sciences, Wuhan 430074, China*

***^5^*** *Key Laboratory of Ecology of Rare and Endangered Species and Environmental Protection (Guangxi Normal University), Ministry of Education, Guilin 541006, China*

***^6^*** *Guangxi Association for Science and Technology, Nanning 530023, China*

**
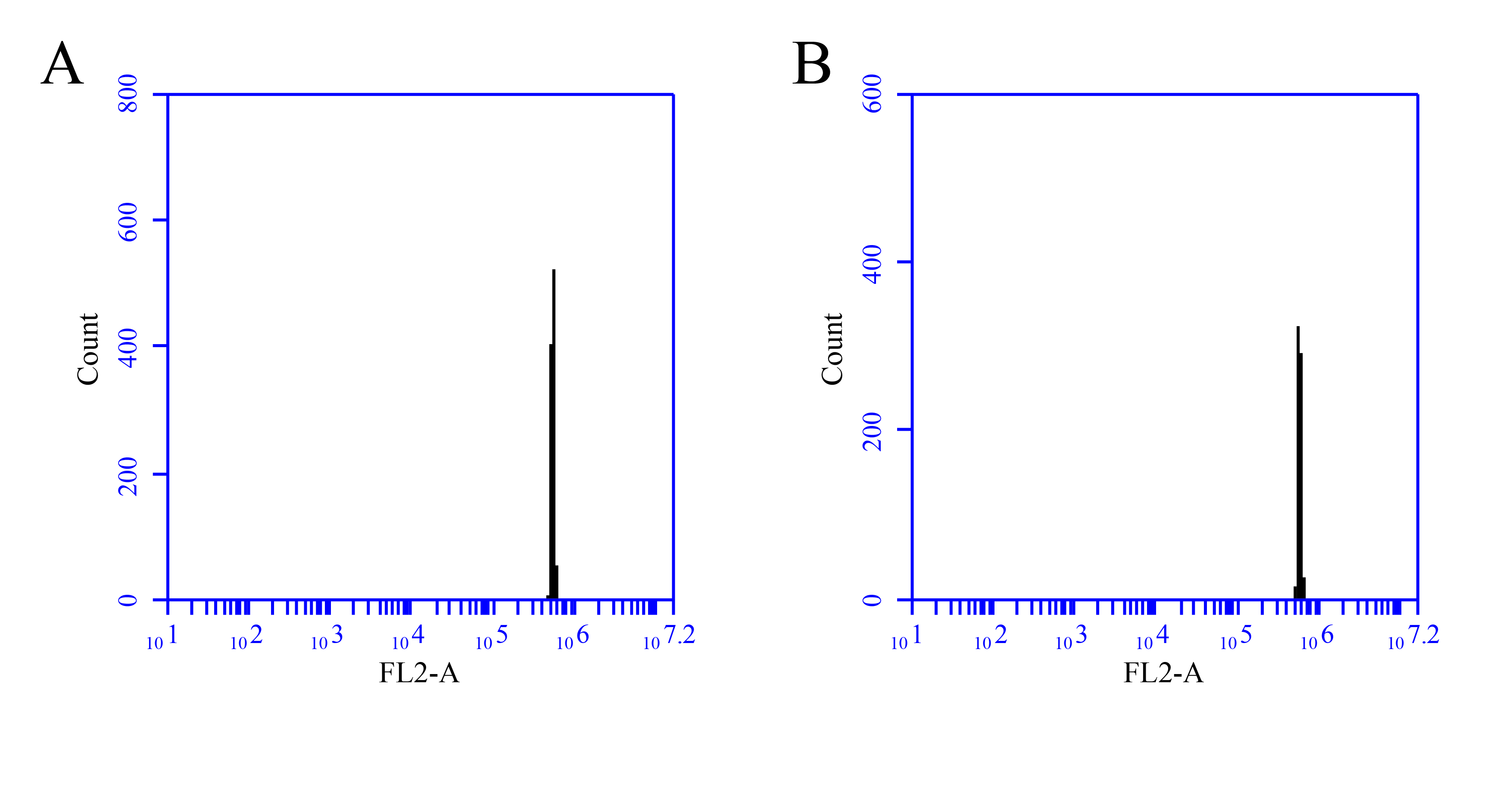
**

**Fig. S1. Results of flow cytometry.**

(A) The genome size of *Nelumbo nucifera* was estimated 808 Mb previously, and the fluorescence was 549,566.70. (B) *Cryptocoryne crispatula*, which fluorescence was 602,867.61, and estimated the genome size to be 886 Mb.

**
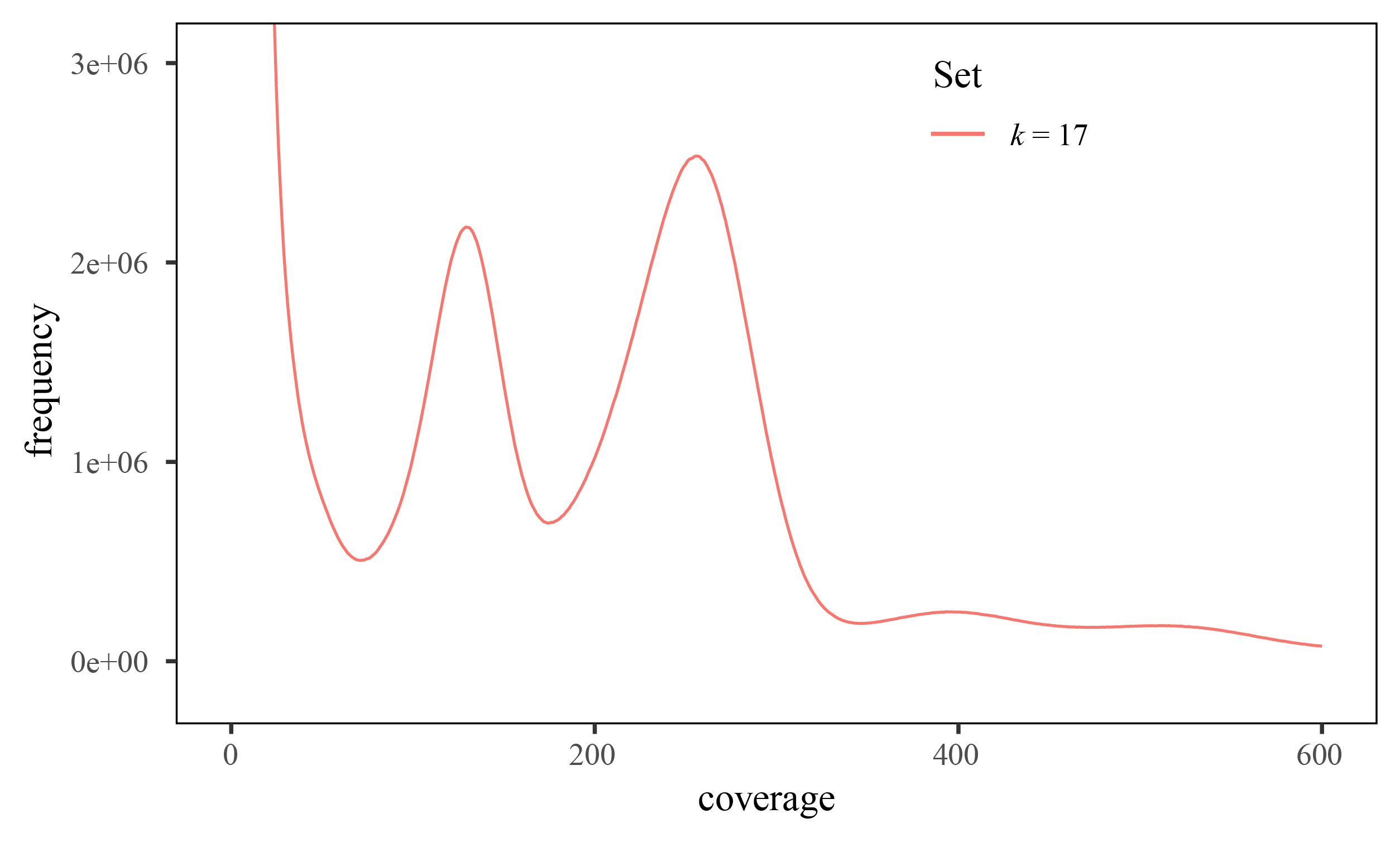
**

**Fig. S2.** **Estimation of *C. crispatula* genome size based on *k-mer*** **= 17 analysis.**

**
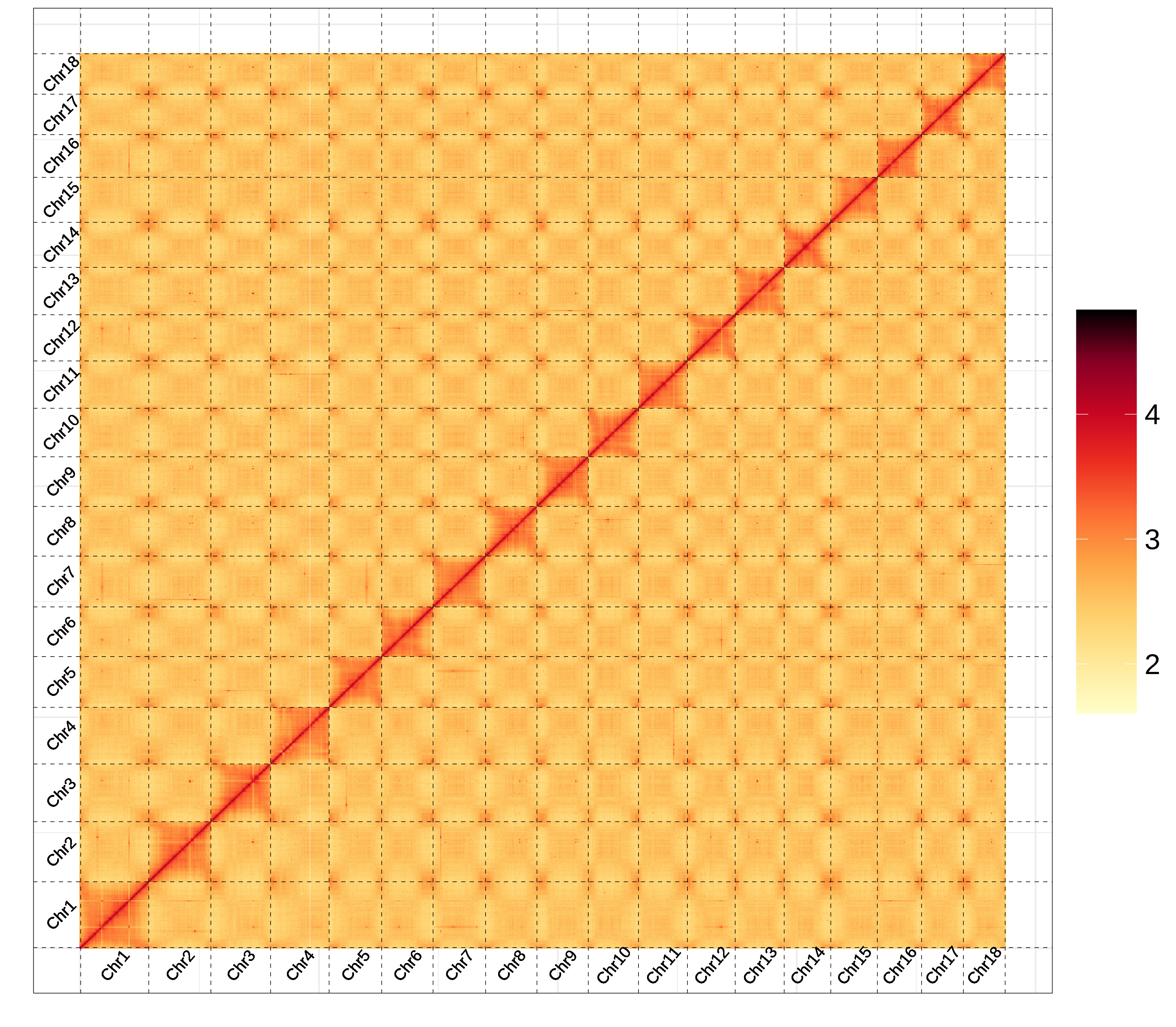
**

**Fig. S3. Hic heat map.**


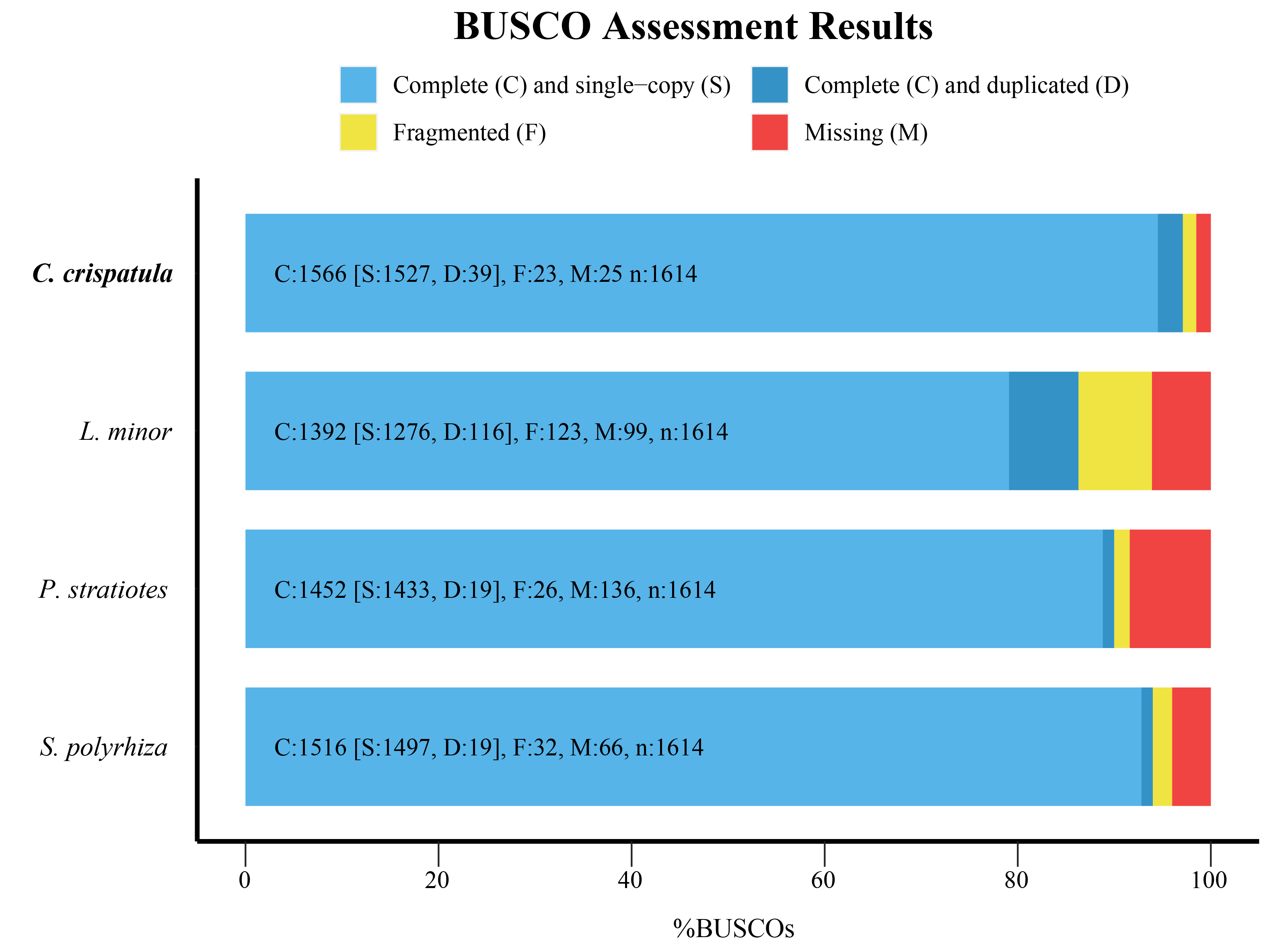


**Fig. S4. Summary of BUSCOs analysis of four freshwater plants genome assembly.**


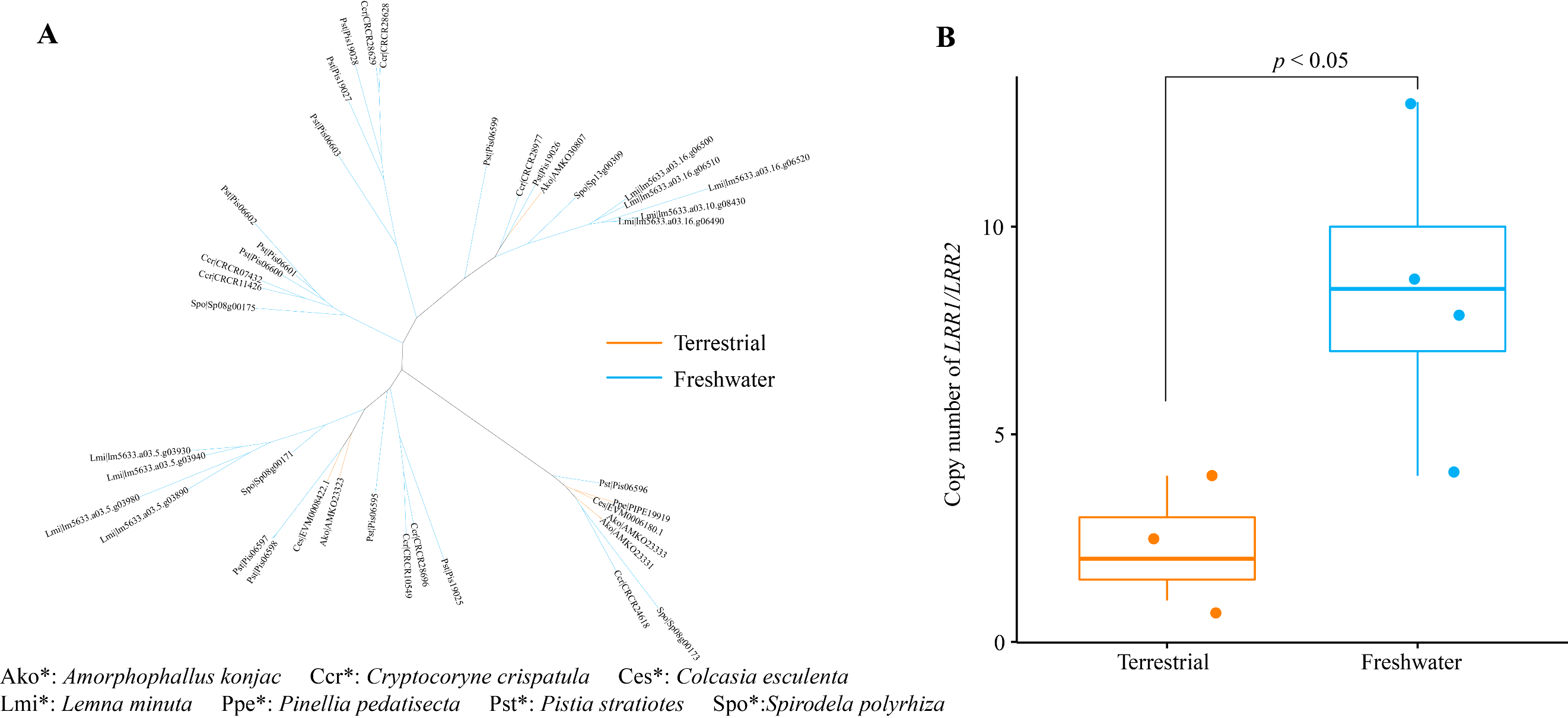


**Fig. S5. Identification of *LPR1/LPR2* in seven species.**

(A) Phylogenetic tree of *LPR1/LPR2* with JTT model using FastTree2; (B) There is a significantly higher copy number of *LPR1/LPR2* in freshwater plants than in terrestrial plants. *p*-value is from Wilcoxon signed ranks test.


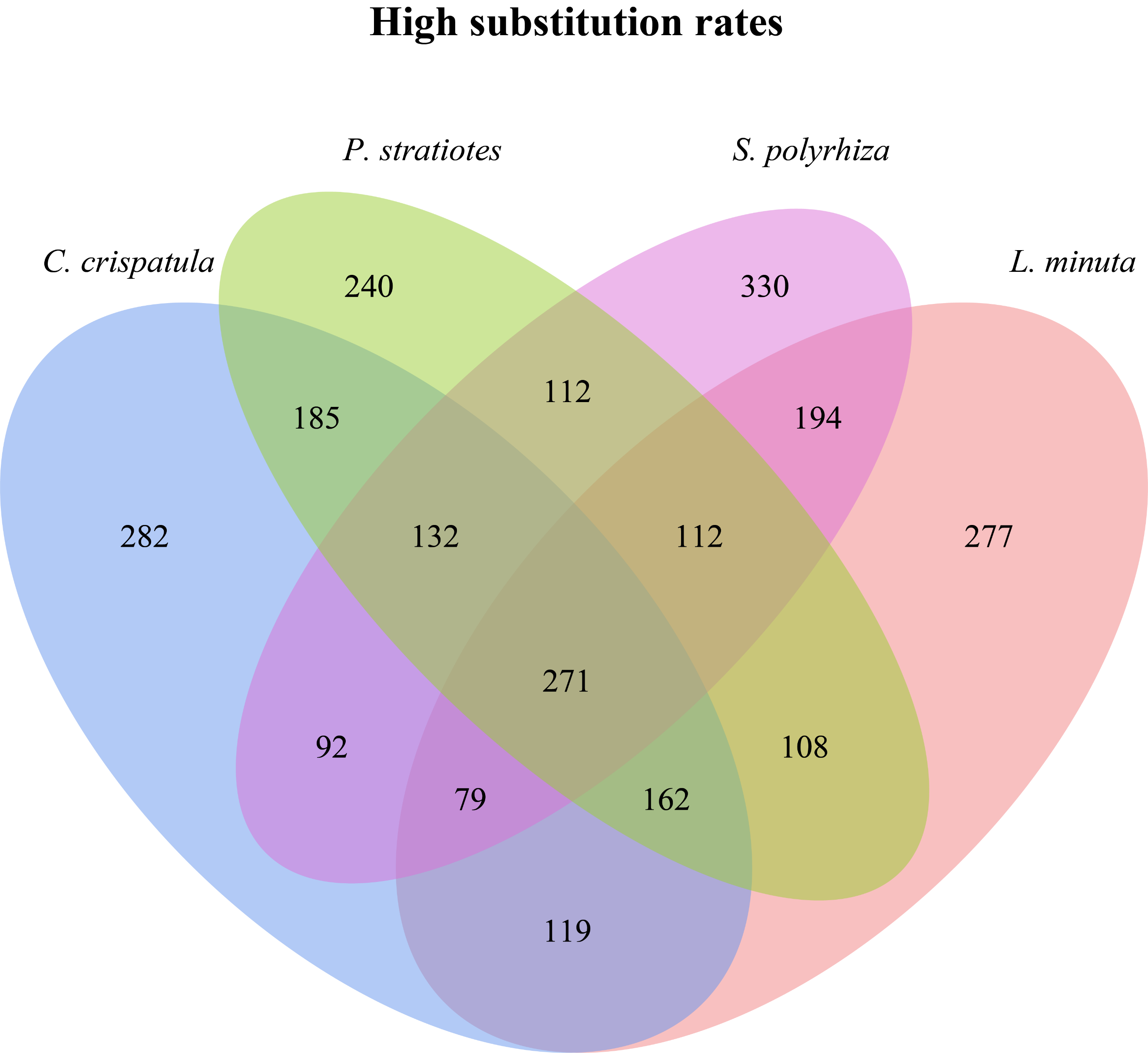


**Fig. S6. The Venn diagram illustrates shared and distinct cluster classes of orthologous genes with “high *dS*” in four freshwater species.**


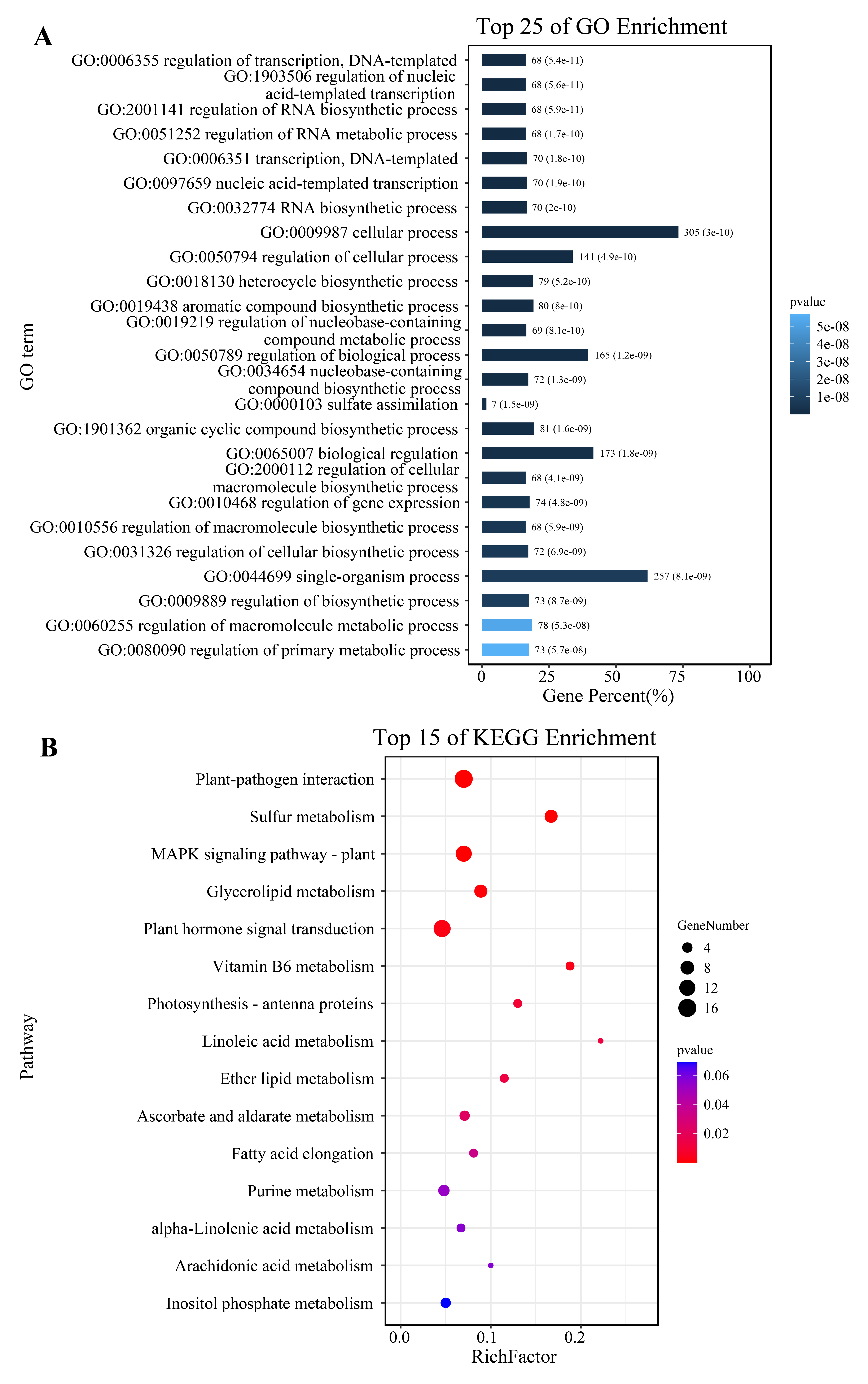


**Fig. S7. The GO and KEGG enrichment analysis for genes with high mutation rate.**

1. GO enrichment analysis; (B) KEGG enrichment analysis.


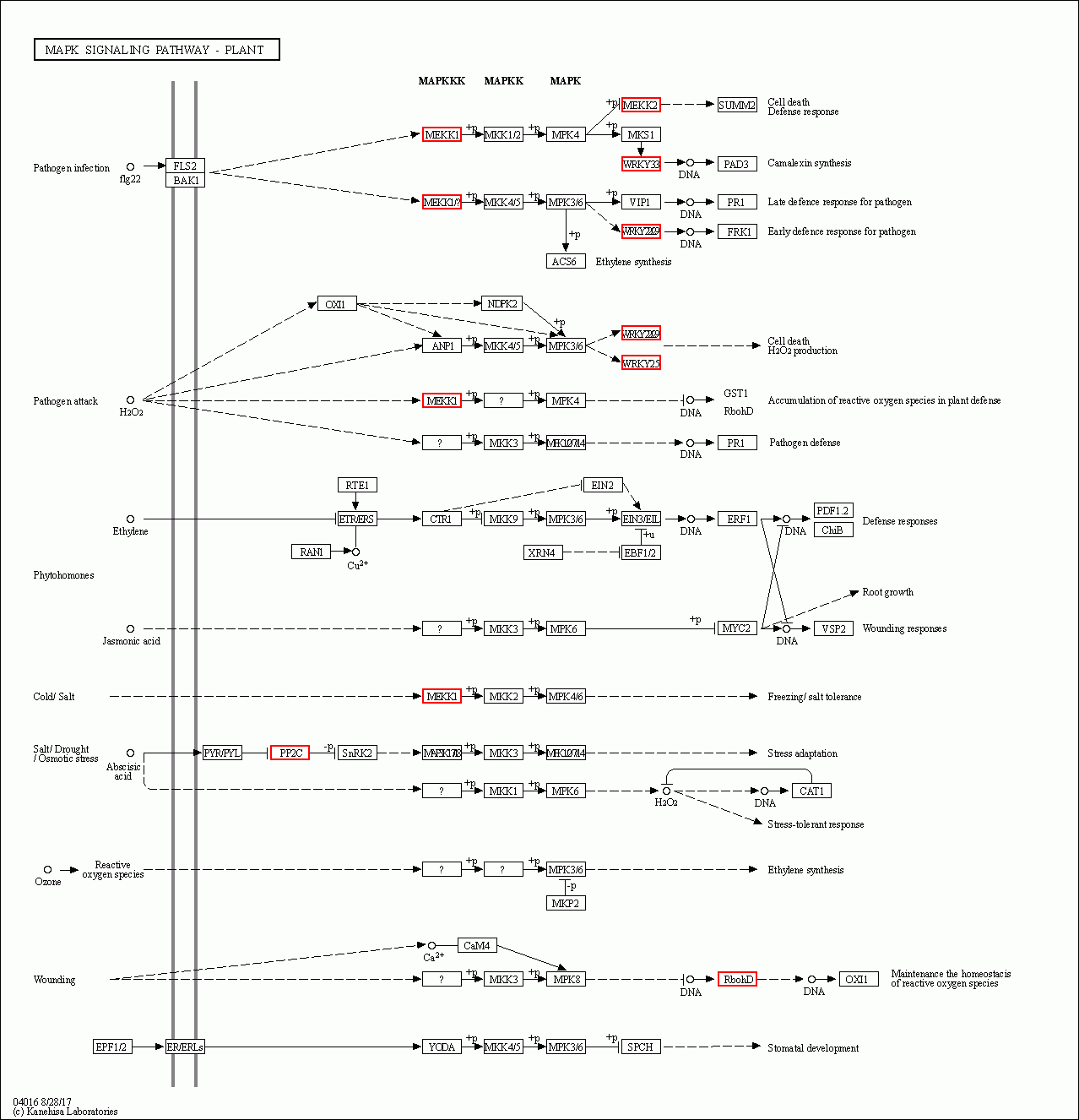


**Fig. S8. The genes with high mutation rate of in** **MAPK signaling pathway – plant pathway of** **four freshwater species.**


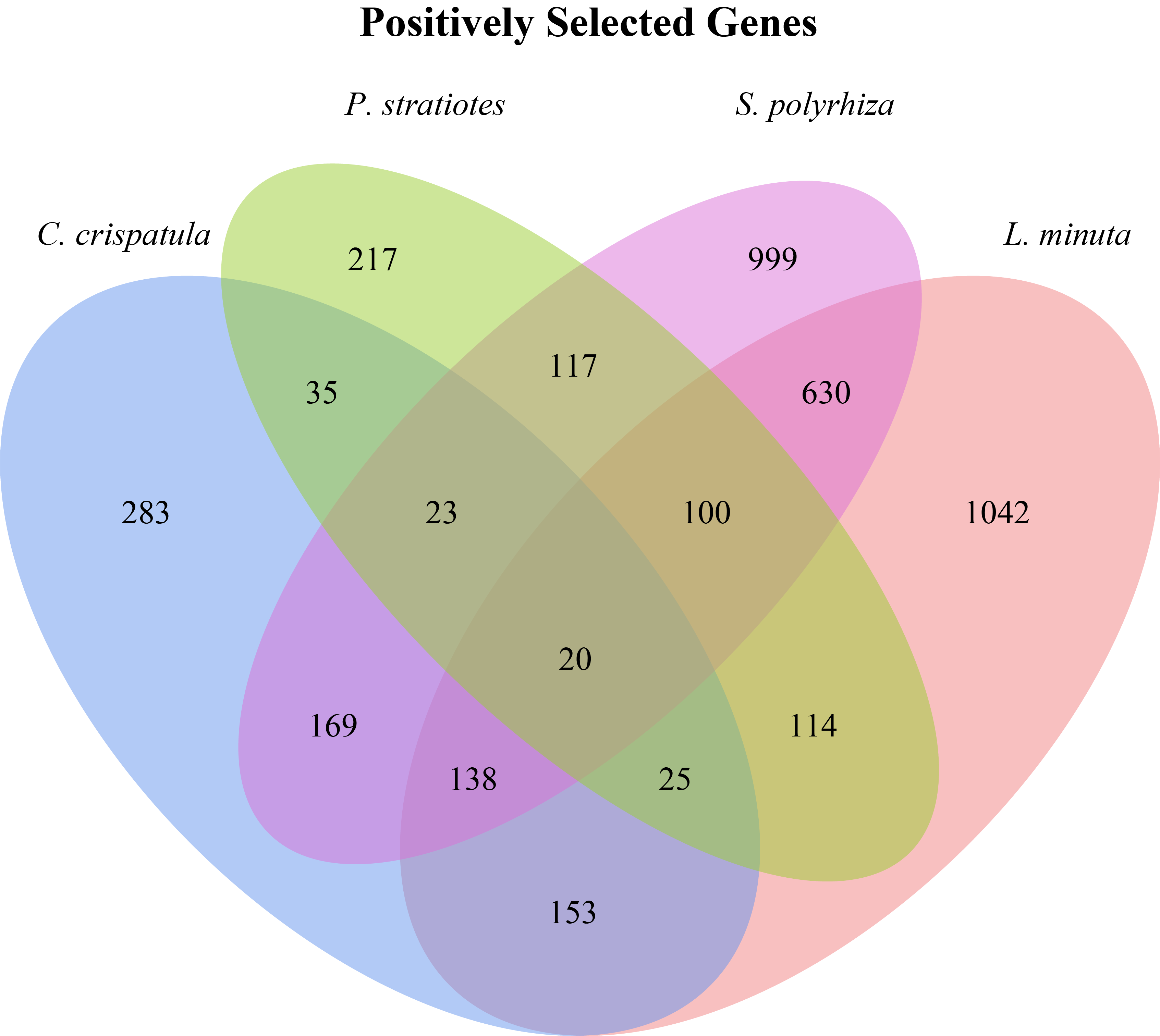


**Fig. S9. The** **identification of positively selected genes in four freshwater species.**


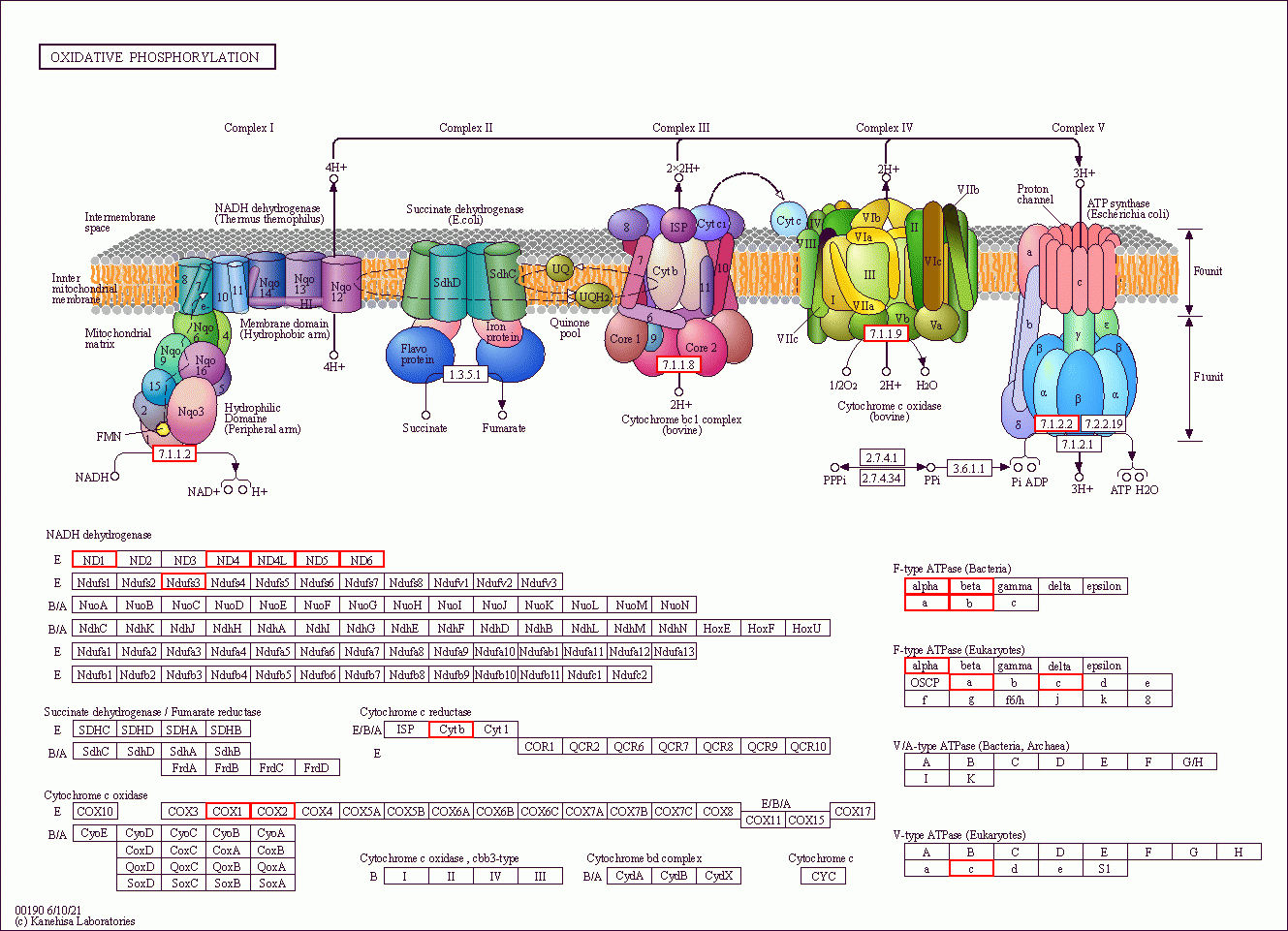


**Fig. S10.** **The submerged plant-specific synteny genes in oxidative phosphorylation pathway.**


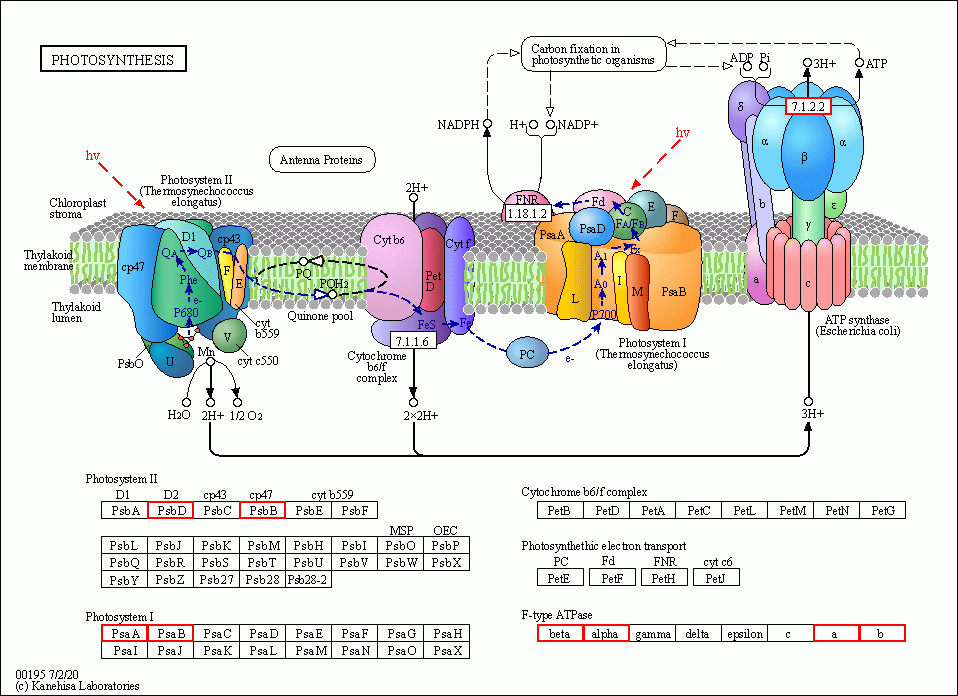


**Fig. S11. The submerged plant-specific synteny genes in photosynthesis pathway.**
